# Variations in Clustering of Multielectrode Local Field Potentials in the Motor Cortex of Macaque Monkeys during a Reach-and-Grasp Task

**DOI:** 10.1523/ENEURO.0047-24.2024

**Published:** 2024-09-26

**Authors:** Florian Chambellant, Ali Falaki, Ian Moreau-Debord, Robert French, Eleonore Serrano, Stephan Quessy, Numa Dancause, Elizabeth Thomas

**Affiliations:** ^1^Unité INSERM 1093, Université de Bourgogne, Dijon Cedex 21078, France; ^2^Département de neurosciences, Faculté de médecine, Université de Montréal, Montréal, Québec H3C 3J7, Canada; ^3^LEAD - CNRS UMR 5022, Université de Bourgogne, Dijon 21000, France

**Keywords:** local field potentials, motor control, motor cortex, reach-and-grasp

## Abstract

There is experimental evidence of varying correlation among the elements of the neuromuscular system over the course of the reach-and-grasp task. The aim of this study was to investigate if modifications in correlations and clustering can be detected in the local field potential (LFP) recordings of the motor cortex during the task. To this end, we analyzed the LFP recordings from a previously published study on monkeys that performed a reach-and-grasp task for targets with a vertical or horizontal orientation. LFP signals were recorded from the motor and premotor cortex of macaque monkeys as they performed the task. We found very robust changes in the correlations of the multielectrode LFP recordings that corresponded to task epochs. Mean LFP correlation increased significantly during reach and then decreased during grasp. This pattern was very robust for both left and right arm reaches irrespective of target orientation. A hierarchical cluster analysis also demonstrated similar changes. In focusing on correlations, our study has contributed new insights to the understanding of LFP signals and their relationship to movement. A sliding window computation of the number of clusters was performed to probe the capacities of the LFP clusters for detecting upcoming task events. For a very high percentage of trials (97.89%), there was a downturn in cluster number following the Pellet Drop (GO signal) that reached a minimum preceding the Start of grasp, hence indicating that cluster analyses of LFPs could contribute to signaling an increased probability of the Start of grasp.

## Significance Statement

The creation of muscular groups also called synergies for accomplishing an action is a well-known feature of motor control. Since the motor cortex plays an important role in creating motor commands, it is only to be expected that such features might also be seen in this brain area. This study is among the first to show that alterations in local field potential (LFP) correlations as a function of task phase can be observed during the reach-and-grasp task by macaque monkeys. The LFPs recorded using multielectrode arrays in the motor cortex showed increased correlations during reach, followed by decreased correlations at the start of grasp. This pattern was robust and present irrespective of which arm was employed or hand orientation.

## Introduction

The optimal functioning of muscular activity requires that they couple and uncouple to the right extent in a timely manner. The breakdown of this coordination very clearly leads to dysfunction of the upper limbs for our daily tasks. An example of this is the excessive muscular coactivation patterns seen during the early stages following stroke. During this state, muscles show poor ability to contract in isolation giving rise to either extensor or flexor synergies (Twitchell, 1951; Brunnstrom, 1970; Baak et al., 2015). In hand functioning, the power grip which requires less finger individuation is favored in these conditions (Li et al., 2003; Xu et al., 2015). At the other end of the continuum are situations of poor interjoint coordination for some movement disorders (Levin, 1996; Astill and Utley, 2008).

Given the importance of correlations in muscular activity, it is natural to expect that we might find this reflected in the neural command structure. Some research groups have found that neural stimulation is able to elicit movements with low dimensional stereotypical patterns. For example, transcranial magnetic stimulation (TMS) in the primary motor cortex can elicit hand movements of low dimensionality with a modular architecture (Gentner and Classen, 2006; Schwenkreis et al., 2007; Bufalari et al., 2010). While these examples of correlation remain expressed at the muscular level, more direct evidence of such clustering at the neural level are also available; e.g., Overduin et al. (2015) demonstrated the presence of low dimensional neural activation patterns during reach-and-grasp in multiunit recordings of the motor cortex of monkeys. Leo et al. (2016) predicted hand postures from corresponding low dimensional representations of fMRI data from the motor cortex.

In the present study, we continue with the effort to characterize this organization of neural commands in association with the reach-and-grasp movements. In contrast to the previous studies on low dimensional activities in the motor cortex using neuronal spiking activity (Overduin et al., 2012, 2015) and fMRI (Leo et al., 2016), we used local field potentials (LFPs). As in the case of the electroencephalogram, it is difficult to draw conclusions concerning the significance and origins of LFP signals, hence limiting their usefulness for understanding underlying mechanisms (Herreras, 2016). However, as a low frequency, extracellular signal, it is known to be relatively robust and less subject to factors such as electrode position (Bédard et al., 2004; Scherberger, 2009). We aimed to investigate the correlations between multielectrode LFP activities in the motor cortex. In particular we wanted to know if they would change over the course of task execution. To this end, we analyzed signals collected from multielectrode arrays implanted in the primary motor cortex (M1) of the right hemisphere as well as in the dorsal and ventral premotor cortex (PMd and PMv, respectively) of both hemispheres of macaque monkeys of a previous investigation (Moreau-Debord et al., 2021).

Few studies have reported on how the correlations of LFP activity in the motor cortex evolves as a function of reach-and-grasp task epochs. Spinks et al. (2008) conducted a study of mean correlation within and between M1 and PMv without examining the evolution of this correlation as movement unfolds. Scherberger et al. (2005) examined correlations between field recordings and single unit spiking activity. Our study moves the field ahead by asking if and how clustering properties between LFP electrodes change during the reach-and-grasp task.

To achieve our aims, we used Pearson’s correlation analyses followed by a hierarchical clustering algorithm to examine in more detail how the correlations between the electrodes within the motor cortex evolved over the course of the reach-and-grasp task. Hierarchical clustering has often been used to examine both physical and functional clustering in the field of neuroscience (Merchant and Georgopoulos, 2006; Boly et al., 2012; Liu et al., 2012; Honig et al., 2021). A more pertinent example is a very recent study by Quarta et al. (2022) where hierarchical clustering on calcium imaging signals was used to investigate neocortical activity during a reach-and-grasp task in mice.

## Materials and Methods

### Experimental model and surgery

The data were recorded in two female rhesus macaque (*Macaca mulatta*) monkeys—Monkey M (5.5 kg) and Monkey S (5.7 kg). Details on the surgical procedures and the behavioral task have been previously published (Moreau-Debord et al., 2021). Briefly, animals underwent craniotomies and durectomies to expose M1 and the lateral premotor cortex in both hemispheres. Multielectrode arrays were implanted in the PMv and PMd of both hemispheres, as well as the M1 of the right hemisphere.

LFP data were collected and sampled at 2,035 Hz and neuronal data spiking data at 24,414 Hz with a Tucker-Davis Technologies acquisition system. The recordings were filtered between 0 and 500 Hz to extract the LFPs while spiking data were bandpass filtered between 100 and 5,000 Hz and sorted offline using Plexon Offline Sorter (Plexon) to obtain spikes times for each electrode.

As the acquisition device was only able to record 256 channels simultaneously, a different subset of electrodes were selected for each recording session (total number of implanted electrodes: 370 electrodes in Monkey M and 448 electrodes in Monkey S). Here, we present results from two sessions recorded in Monkey M and two sessions in Monkey S during which neuronal recordings were obtained from most of the implanted brain areas.

### Behavioral task

**Figure 1.**
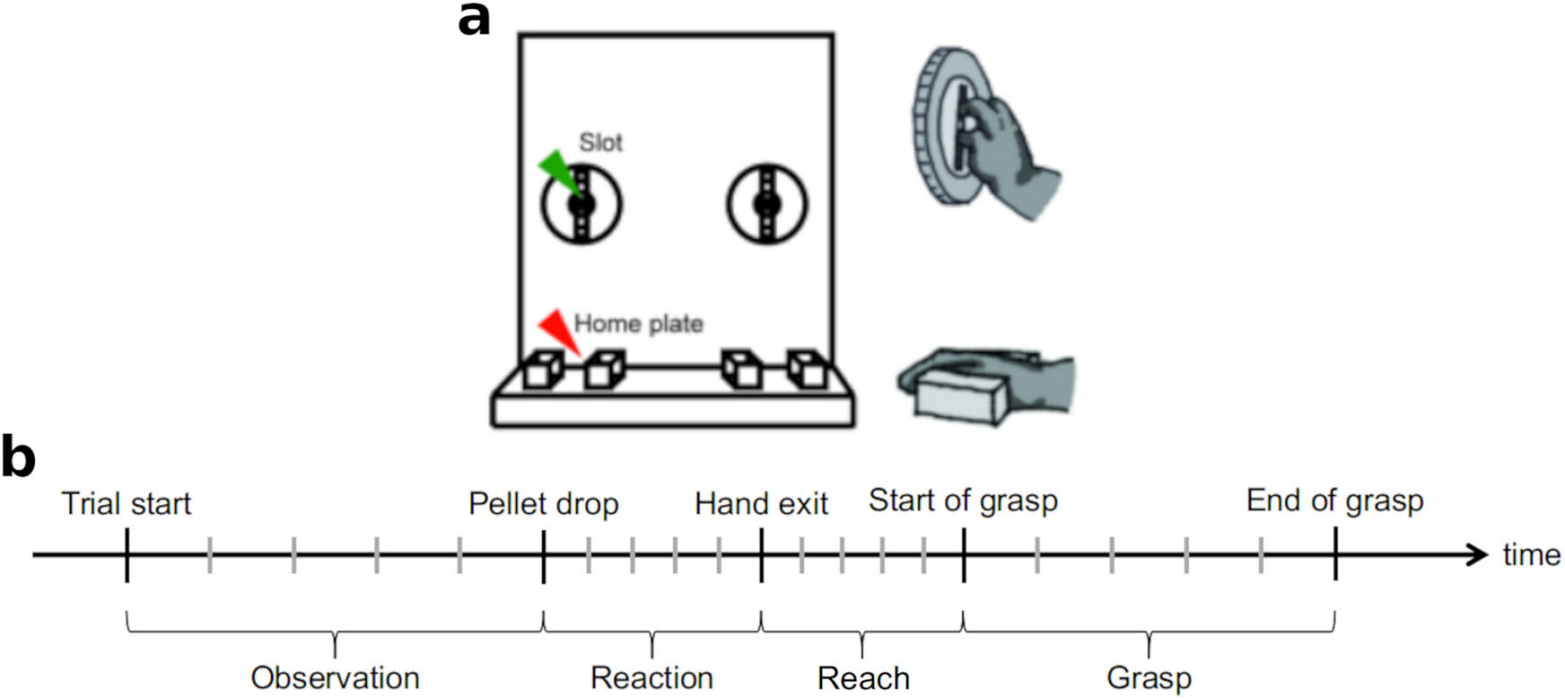
***a***, Experimental apparatus (adapted from Moreau-Debord et al., 2021). ***b***, Trial segmentation and events. More details on the manner in which each epoch was defined can be found in the Materials and Methods, Behavioral task.

The monkeys sat on a custom-made primate chair placed in front of a food pellet dispenser. The chair was equipped with an opening for the mouth and two removable panels, one at each side of the chair, allowing the monkey to use one hand to withdraw the food pellets and eat them. Either the left or the right opening for the arm (depending on the trial block) was available for the monkey to pass its hand through and retrieve the food pellet dropped in a well behind a slot (1.3 × 5.5 cm) located ∼10 cm below the shoulder height and 20 cm from the monkeys. Depending on the trial block, the slot had either a horizontal or vertical orientation, hence forcing the animal to appropriately supinate or pronate the hand and obtain the pellet using a precision grip with the thumb and index (see Fig. 1*a* for example of vertical grasp).

Each trial began after the animal placed its hand on the home plate located 15 cm below the pellet slot. The presence of the hand was detected by an infrared laser sensor embedded in the home plate and marked as “Trial start.” After a random delay ranging from 800 ms to 2 s, a pellet was dropped into the well (Pellet drop) using a pellet dispenser (80209 Pellet Dispenser, Campden Instruments). The sound resulting from the pellet drop served as a GO cue for the monkey. Following the pellet drop, the animal had 2 s to remove its hand from the home plate (Hand exit) and Reach toward the pellet. The time when the animal entered its hand in the slot to grasp the pellet (Start of grasp) and when the hand was removed from the slot (End of grasp) were detected by a second infrared laser sensor embedded at the entrance of the slot. The monkey brought the pellet to its mouth and placed its hand back on the home plate to initiate the next trial with an intertrial interval of 3 s. The animals repeated the task 25 times for each hand and slot orientation (block randomized). See Figure 1*b* for an indicative timeline of the task.

### Preprocessing

Custom scripts written in Matlab (The MathWorks Inc., 2021) and the Fieldtrip toolbox (Oostenveld et al., 2010) were used to process the neural data. Prior to performing the correlation and clustering analyses, neural data were preprocessed to remove bad trials as well as potential noise and artifacts. Trials for which the animal's Reaction time was inferior to 200 ms (the monkey successfully predicted the pellet drop), the Reach duration was above 350 ms, or the grasping lasted <200 ms (the monkey was unsuccessful in retrieving the pellet in a single try) were discarded. This represented 5.2% of the trials. Following the rejection of bad trials, the LFP data were cleaned using a low-pass Butterworth filter (200 Hz, sixth order) followed by the removal of 60 Hz noise using a notch Butterworth filter with band stop limits of 59 and 61 Hz. Irregular bursts and low frequency irregular oscillations were removed using independent component analysis (ICA; Bell and Sejnowski, 1995; Whitmore and Lin, 2016). Since we collected LFP data in blocks of 32 electrodes using Omnetics connectors, the ICA procedure was applied to the data collected by each individual connector to reduce spreading signal cancellation. When the ICA procedure failed, i.e., >10 components contained noise, data from the entire connector was discarded. Following these procedures, electrodes with outlier LFP activity, i.e., greater than the mean ± 2 SD were also removed. Further preparation of the LFP data consisted of removing effects of neuronal spiking activity. Recoded information on the spike timings were used to replace the data of an electrode, corresponding to the spiking activity, from 1 ms before to 2 ms after the spike time by linear interpolation between these two points (Waldert et al., 2013). The number of trials and electrodes for each area for the two sessions of the two monkeys can be seen in Table 1.

**Table 1.** Summary of the different sessions analyzed in the study

Session	Number of trial conditions				Number of electrodes				
	Left hand horizontal grasp	Left hand vertical grasp	Right hand horizontal grasp	Right hand vertical grasp	M1	lPMd	rPMd	lPMv	rPMv
M14	26	23	22	23	18	84	29	-	-
M17	23	25	23	22	-	61	41	46	28
S19	25	24	25	20	24	21	22	29	73
S26	25	24	25	25	58	24	-	76	54

**Figure 2.**
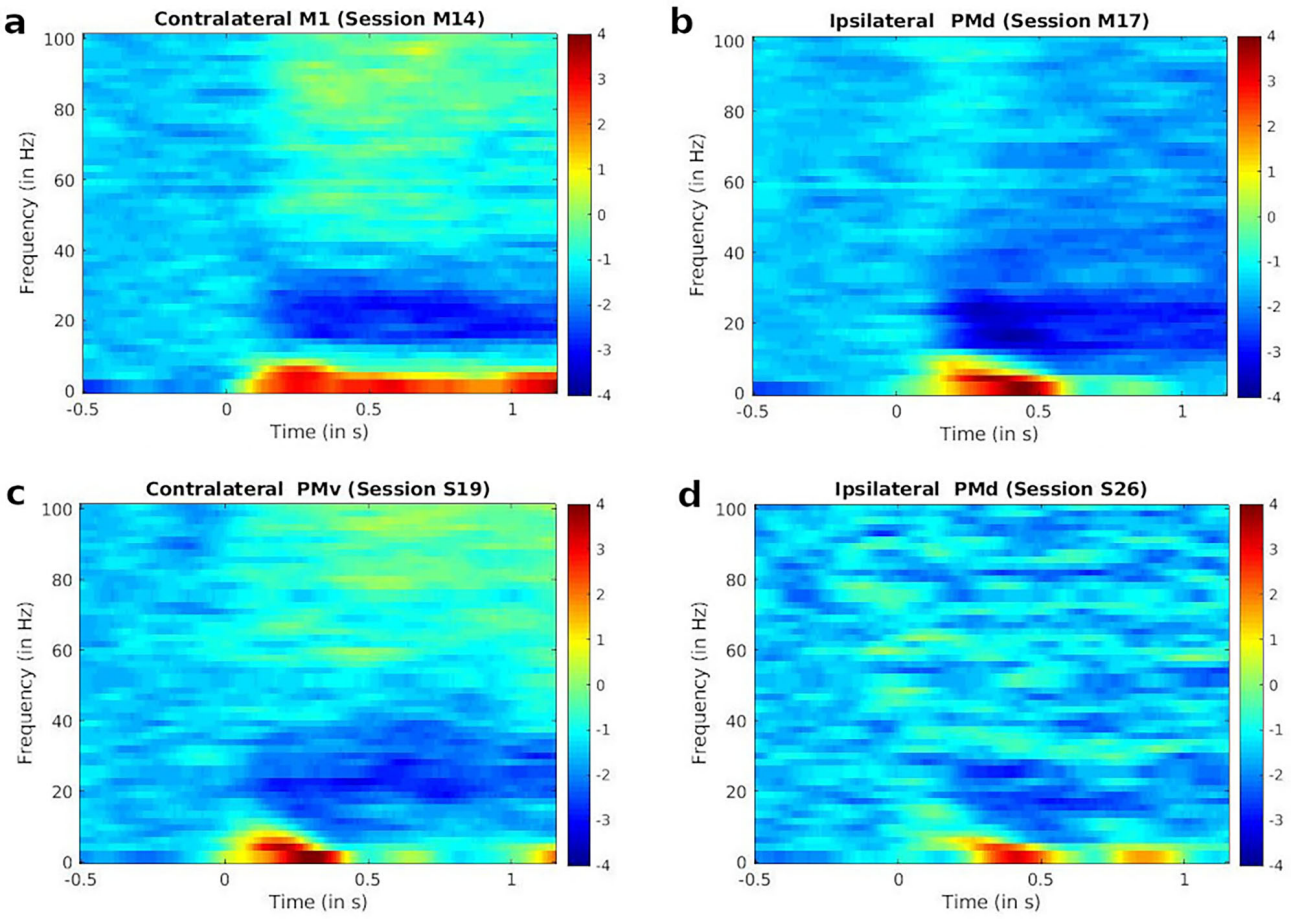
Examples of spectrograms of the LFP activity, during different sessions, aligned at Pellet drop (Time = 0; Pellet drop served as the GO cue) averaged across all trials and electrodes for the indicated movement type (ipsilateral or contralateral) and brain area of the session.

The general steps for computing the spectrogram were done following Waldert et al. (2015) and Menzer et al. (2014). These steps were removing noise from the signals, selecting appropriate channels and trials, computing the spectrograms, and finally, normalizing the spectrogram for each trial and frequency using a baseline from the observation phase. More specifically, we used a single taper method with a 400 ms sliding Hann window and 10 ms time steps (Fig. 2).

It can be observed in Figure 2 that the highest spectral powers were observed in the delta band (∼0–4 Hz).

### Correlation

The similarity in the spectral power of the LFP activities over the course of the reach-and-grasp task was first analyzed using a correlation analysis. Each trial was divided into four phases based on the events of the trial, i.e., Observation, Reaction, Reach, and Grasp (Fig. 1*a*,*b*). In all cases, correlations were computed across all electrodes irrespective of the area of the motor cortex in which they were implanted. For each trial, average correlations was obtained from the absolute values of the pairwise correlations between all electrodes of the trial; i.e., correlations were processed without taking their sign into account. In cases where we were displaying the correlations from a session, the correlation values were averaged across trials. While in Figure 4*a*,*b*, we display the correlation at all the frequencies, the other analyses of the paper were focused on the delta frequency band, so that the final correlation values analyzed were an average of correlations across the delta band.

As there was a difference in trial numbers for some of the sessions, we undertook a comparison of the average correlations to verify if they had an effect on the variable. This was not likely to be the case with such numbers of correlation pairs—not only arising from trial numbers but also number of electrodes (minimum number of 131). As the biggest difference in trial numbers was observed for the session S19, we undertook the comparison for this session. In Table 2, we can see the results of a *t* test comparison between the average correlations using the 20 trials from the right vertical grasp, with the average correlations from the right horizontal grasp. In the case of the latter, the trial numbers were increased from 20 to 25. For all cases with <25 horizontal trials, they were randomly picked from the pool of trials. The test showed that the changes in trial number did not introduce any big changes in correlation values.

**Table 2.** Student's *t* test comparison of correlations between a fixed number of right arm vertical grasp trials and a varying number of right arm horizontal grasps

Trial numbers		*p* value
Right arm vertical grasp	Right arm horizontal grasp	
20	20	0.60
20	21	0.60
20	22	0.50
20	23	0.56
20	24	0.52
20	25	0.51

The comparisons showed that the differences in trial numbers did not lead to significant differences in correlation.

### Hierarchical clustering

**Figure 3.**
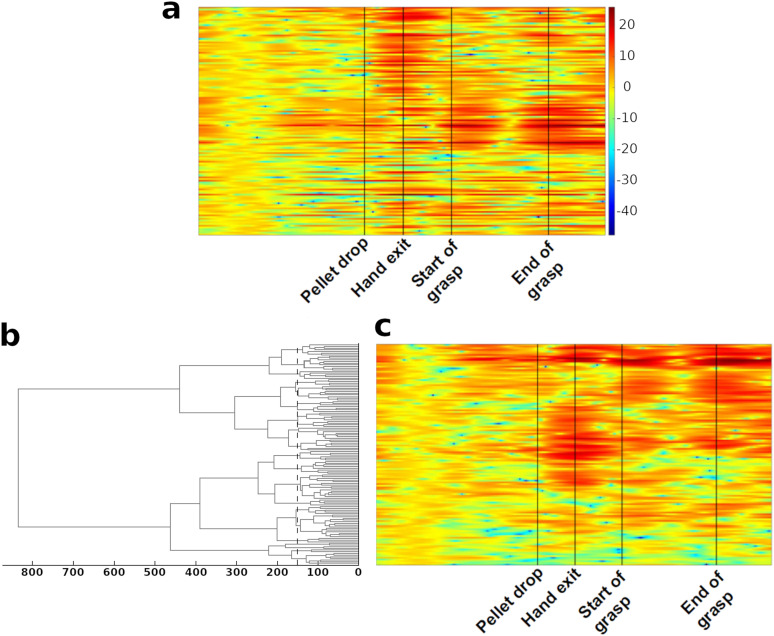
Illustration of hierarchical clustering technique. ***a***, LFP spectral power at 2.5 Hz (scale on the right) for all the electrodes from a single trial of Monkey M. Each horizontal line corresponds to the LFP spectral power of one recorded electrode for the entire trial. The trial epochs are indicated by vertical black lines. ***b***, Dendrogram obtained by using hierarchical clustering on the data of ***a***. The dashed black vertical line indicates a distance of 150 (a.u.). This line was used as the point at which cluster numbers were counted. ***c***, Data of spectrogram in ***a*** reorganized to follow the dendrogram presented in ***b***. The clustering algorithms was applied to the entire trial and led to the clustering of information from electrodes with correlated spectral power.

A finer analysis of the proximity of activities between the LFP electrodes was obtained using a cluster analysis. In this method, a distance measure is used to cluster together similar data points. The idea of hierarchical clustering (Ward, 1963; Hastie et al., 2009) is to create a hierarchy of similarity between data points. This hierarchy is usually presented as a dendrogram, with each branch containing data points which are close. The distance between any two clusters is represented by the length of the line holding apart the clusters (Fig. 3*b*). The primary method used to compute the distance between data points was correlation.

The process began with the computation of a matrix of absolute correlations between each pair of electrodes. The correlation pattern of the two closest electrode pairs were then “fused” together, and a new correlation matrix was obtained with the “fused” data. This process was repeated until all the points were fused together. Following this, a distance criterion was used to count the number of clusters at a fixed point. Each potential cluster was composed of a branch and all its “leaves” at this distance (Fig. 3*b*). The cutoff distance for estimating cluster number was fixed by observing the point at which there was a sharp drop in cluster number (this is similar to the elbow method used with PCA to count the number of components). This distance criterion was set during the Observation phase. This criterion was then used to compute cluster numbers for each trial and subperiod of each session. As we were more interested in the changes of cluster number with the progression of the reach-and-grasp task rather than the actual number of clusters itself, the measure that was finally used to follow the similarity in LFP activity was normalized cluster number; i.e., the cluster number at Pellet drop was set to 1.

The hierarchical clustering was applied in two ways. In one case, the entire trial was divided into subperiods, and the cluster numbers were computed for each subperiod (Fig. 1*b*). In the second case, the cluster numbers were computed using a sliding window, hence giving us an instantaneous cluster number. In both cases, the correlation matrix of the LFP signals in the delta band during the time period of interest was computed followed by the application of the hierarchical clustering method to the matrix.

### Statistical tests

Repeated-measures ANOVA and Student's *t* test were used as the main statistical tests. In the case of using ANOVA, post hoc analysis was conducted using Tukey's HSD test. The data were checked for assumptions of normality and sphericity and were transformed accordingly to satisfy statistical assumptions of normality. If the data did not respect the assumption of sphericity, *p* values were corrected using the Greenhouse–Geisser correction, and Holm's test was then used for post hoc analyses. For all statistical tests, *p *< 0.05 was considered as the statistically significant value.

## Results

In the sections below, we present the results from the correlation and clustering analyses which were performed on the LFP data during the reach-and-grasp task by two monkeys during their two sessions. The computation of correlations and clusters was done across several motor areas without taking into account the specific area of the motor cortex in which the electrodes were found. This is in keeping with the idea that correlation and cluster formation in the brain would reflect what has been observed in human behavioral studies, i.e., varying degrees of coupling between the shoulder, upper arm, lower arm, and hand, during reaching and grasping movements (Jeannerod, 1984; Wallace et al., 1990; d’Avella et al., 2008; D’avella and Lacquaniti, 2013). The analyses of the study were focused on the delta band as we found the largest modulations of spectral power in this band following pellet drop as seen in the spectrograms of Figure 2. Others have also reported the link between activity in the delta band of the motor cortex and arm movement (Rickert et al., 2005; Zhuang et al., 2010; Milekovic et al., 2015; Waldert et al., 2015).

### Pairwise correlation of LFP spectral power in the delta band

**Figure 4.**
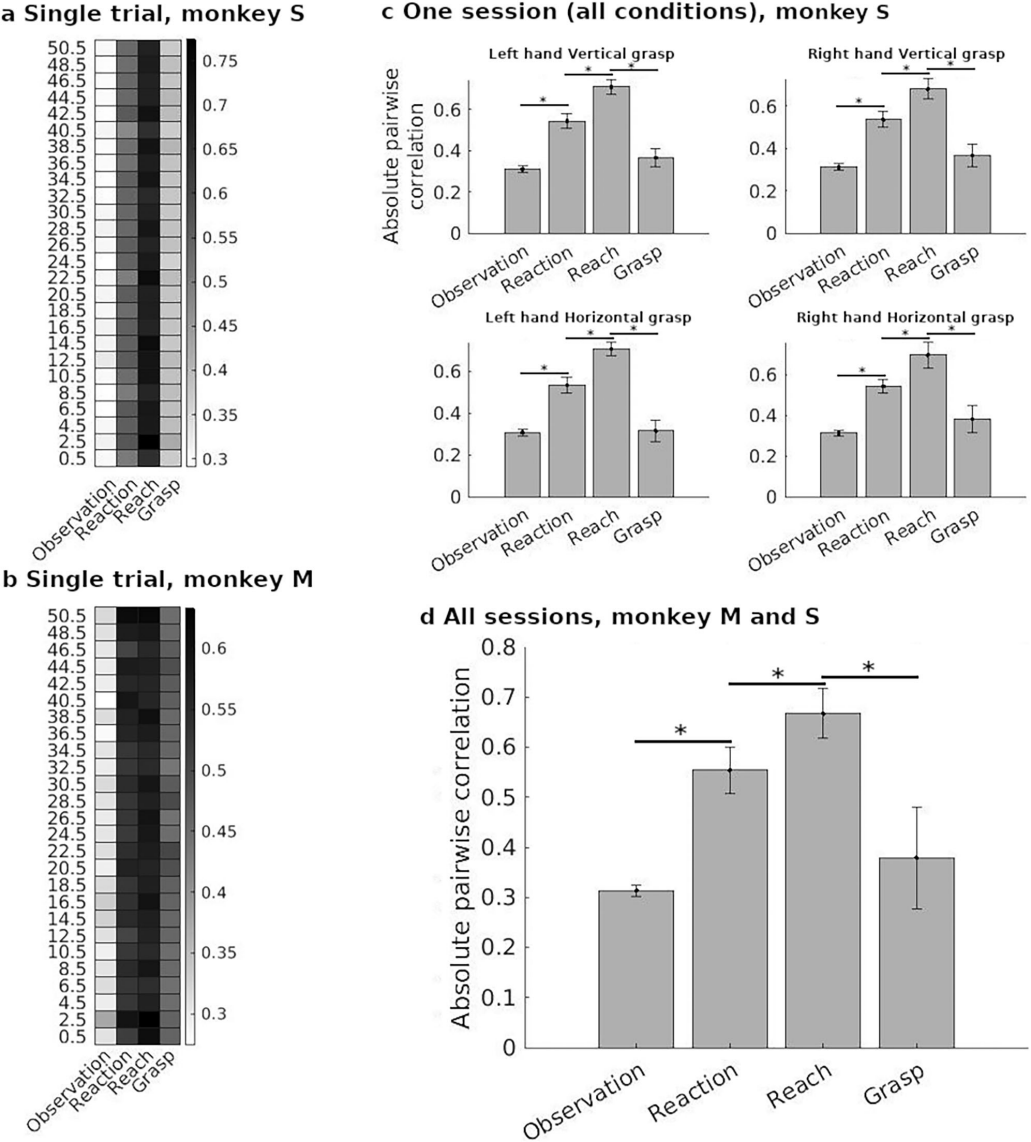
***a***, ***b***, Heatmap of the average absolute pairwise correlation of LFP spectral power between electrodes at different frequencies during each phase of the movement for one trial of Monkeys S and M. ***c***, Average absolute pairwise correlation of spectral power for the delta band for all the trials in one session for Monkey S under different trial conditions. Error bars indicate the variance between trials. ***d***, Average of the absolute pairwise correlation in the delta band for all trials and sessions performed by the two monkeys (two sessions per monkey). Note that this included different pointing conditions. Error bars indicate the variance between trials.

The correlation analyses provided us with a picture of the evolution of similarities between the recorded multielectrode LFPs over the course of the reach-and-grasp task. The correlation analysis was first done by looking at the spectral power for all frequencies of one trial of Monkeys S and M (Fig. 4*a*,*b*, respectively). We then moved on from there to narrowing the investigation down to the delta frequency (frequency with the highest spectral power) for all the trials in one session for Monkey S (Fig. 4*c*). The session included different pointing conditions—the arm used and target orientation. This was done in order to see how generalized the patterns of correlations transitions were for several types of reach-and-grasp. Finally in Figure 4*d*, we computed the averages for all the trials for all task conditions of both sessions for both monkeys in order to test statistical significance. Below, we provide more details on each figure.

Figure 4*a* displays the correlation values for one trial performed by Monkey S, during which the left arm was used to perform a horizontal grasp movement. The values shown are the averages of the absolute values of pairwise correlations in the LFP spectral power at each frequency between all the recorded electrodes (see Materials and Methods). The LFP correlations between the different task epochs were significantly different for this representative trial (repeated-measures ANOVA, F(2.27,56.74)=2,837.65,p<0.001, Greenhouse–Geisser correction for sphericity). More specifically, as compared with the Observation period, there was an increase in the average absolute pairwise correlation during the Reaction time with further increase during the Reach phase. Finally, the average absolute pairwise correlation decreased during the Grasping phase. The correlation changes during all these task epoch transitions were found to be significant using post hoc analyses (Holm's test, *p* < 0.001 for all transitions). The correlation transitions were similar for the single trial of Monkey M at all frequencies as seen in Figure 4*b* and were also statistically significant (repeated-measures ANOVA, F(2.49,62.11)=1,061.96,p<0.001, Greenhouse–Geisser correction for sphericity).

The results displayed in Figure 4*a* are obtained from one trial of one type of reach-and-grasp, namely, a left arm reach for a horizontal grasp. However, the monkeys in this study also performed trials with right arm reaches and vertical grasps. In Figure 4*c* we examine the profile of correlation transitions for the same Monkey S under these different task conditions. In contrast to Figure 4*a*, in Figure 4*c* we focused on the spectral power of the delta band. We first computed the pairwise correlations between the electrodes of single trials, before averaging the correlations over the pertinent trials. Each figure for the different pointing types in Figure 4*c* is the average of all the trials with the same task conditions in that session. Similar profiles of LFP correlation increases and decreases were seen in the different types of reach-and-grasp task conditions for Monkey S.

A final test was done to examine the statistical significance of these transitions for both Monkeys S and M together. As in the case of Figure 4*c*, pairwise correlations were computed for single trials before any averaging. The histogram from this data for all the trials, irrespective of task type, for both monkeys, can be seen in Figure 4*d*. Once again, the correlations between the Observation, Reaction, Reach, and Grasp phases were found to be significantly different as demonstrated by a repeated-measures ANOVA ( F(1.74,671.30)=17.53,p<0.001, Greenhouse–Geisser correction for sphericity). Post hoc analyses showed that there was an increase in correlation upon the transition to the Reaction and Reach phases while the shift to the Grasp phase was accompanied by a decrease in mean absolute correlations (Holm's test, p<0.001 for all transitions).

### Cluster analysis on the LFP spectral power in the delta band

We then applied the cluster analysis to the LFP spectral power in the delta band. This was done in order to obtain a finer grained picture of correlations between the electrodes. We first make a qualitative report that demonstrates how the clustering is not restricted between the electrodes of one area. This is followed by a more quantitative analysis in which we follow the cluster number as a function of task epoch.

#### Clustering of LFP activities between and within areas of the motor cortex

**Figure 5.**
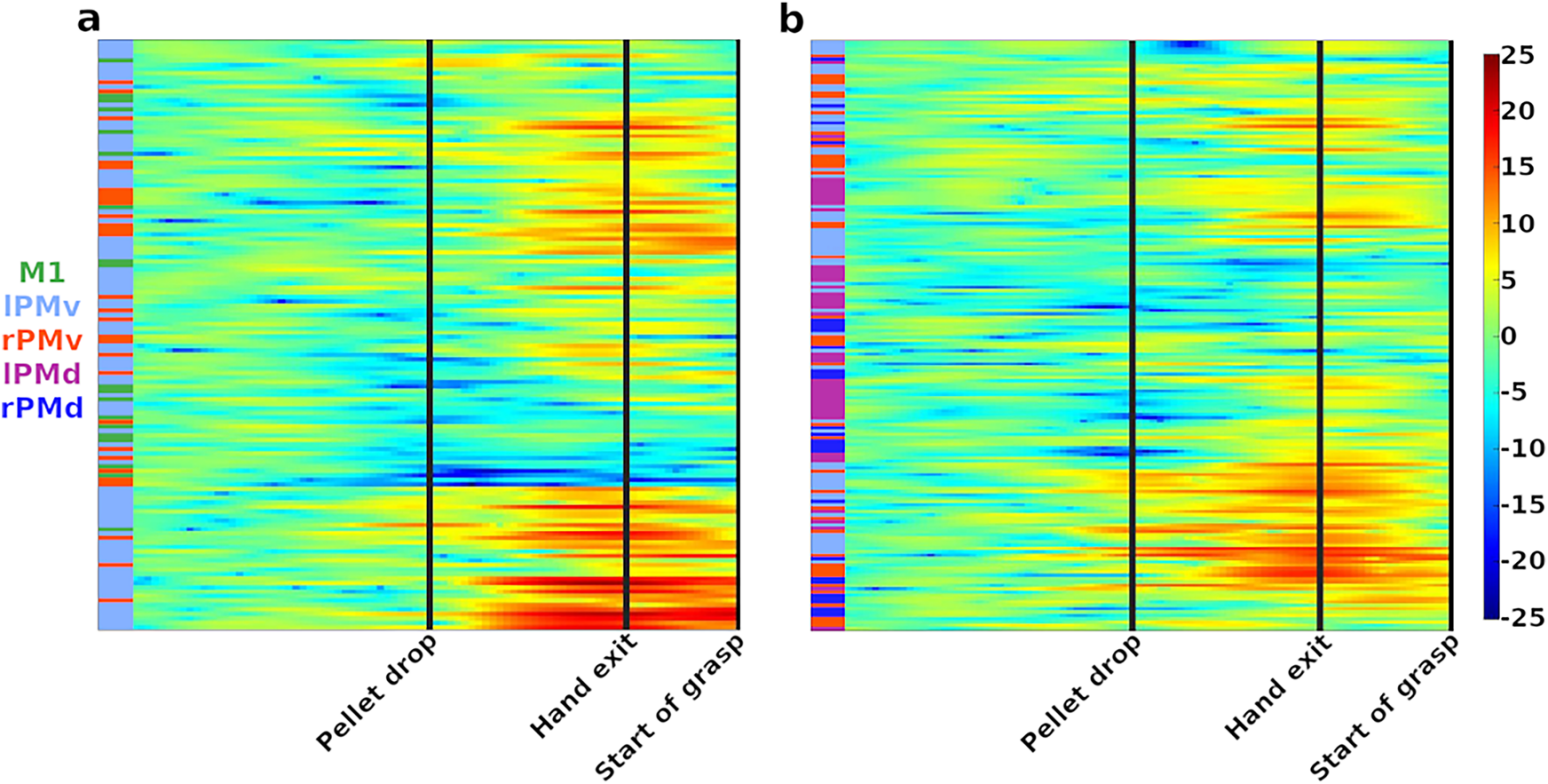
Clustering of the activity in delta band recorded from all the electrodes during one trial of Monkey M. As in Figure 3, each line shows activity recorded from one electrode during the trial. The area of origin of the electrode is color coded by the column on the left of each graph (color legend on the left). It is to be noted that clusters are not restricted to a particular area of the motor cortex and contain LFPs from different regions. The black lines indicate the timing of the task epochs. ***a***, ***b***, are from two different sessions of Monkey M.

The spectrograms of Figure 5 were obtained by applying the cluster analysis to two trials of Monkey M. Figure 5, *a* and *b*, comes from two different sessions of Monkey M. The clustering algorithm was applied to the entire trial. We can see in this figure that while electrodes from a same region often had similar activity (indicated by long patches of the same color in the left column coding for the region of origin of the electrodes), electrodes from different areas could also have correlated activity (indicated by the mix of color in this column). This highlights the idea that the reach-and-grasp movement is accompanied by inter- as well as intraregional correlations.

#### Transitions in cluster numbers as a function of task epoch

**Figure 6.**
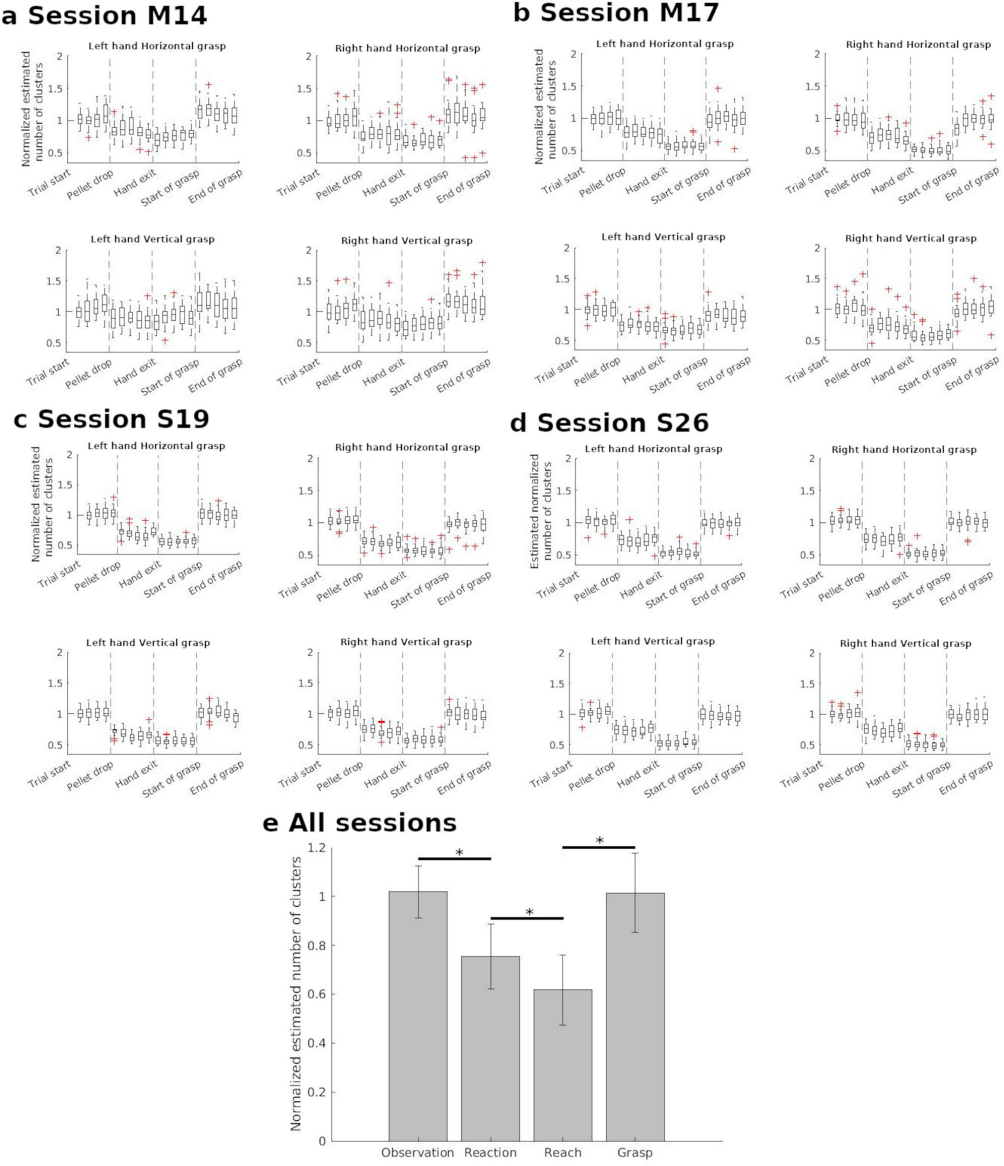
Average normalized cluster number in the delta band as a function of task epochs. Clusters were found using correlations. ***a***, ***b***, For all the trials of the same task conditions for Monkey M. There are trials from two sessions presented. ***c***, ***d***, For all the trials of the same task conditions for Monkey S (over two sessions). ***e***, For all the trials of both sessions of both monkeys. In this case, the average normalized cluster number for each task epoch is the average of the values from the five subperiods. Error bars indicate the variance.

Using absolute pairwise correlations of activity in the delta band provided us with a general idea of the similarities in the recorded LFP activities during the whole reach-and-grasp task. However, in keeping with the notion of synergies between muscles formed during a movement, we wanted to have a more detailed picture of grouping patterns in the LFP activity as the task progressed. We did this by using the hierarchical clustering method described in the Materials and Methods. For this we increased the temporal precision of the analysis by using smaller temporal subsections for the clustering analysis. We divided each of the previously defined task epochs into five equal subperiods and computed the cluster number in each subperiod. Figure 6 presents normalized cluster numbers (see Materials and Methods) as a function of task progression in these subperiods.

As in the case of correlation analyses, we first show the number of clusters of one session for each monkey (Fig. 6*a–d*). Each figure displays the evolution in number of clusters as a function of trial epochs. The titles indicate the task conditions for each figure. They show that for both monkeys, independently of the arm used and grasp orientation, there was a relatively similar profile of clustering transitions. First, there was a decrease in the estimated number of clusters at pellet drop, followed by a smaller drop at Reach onset before going back close to baseline values during grasp. Such transitions in normalized cluster numbers are coherent with the results of the correlation analyses (Fig. 4). Increased averaged pairwise correlation would indicate greater similarities across the activity of different electrodes which in turn would result in a smaller number of clusters.

To confirm the statistical significance of these transitions, we used the data obtained across both sessions of the two monkeys. Figure 6*e* displays the averaged normalized estimated cluster number during each of task epochs across all the sessions for both monkeys. Unlike Figure 6*a–d*, there is only one value of normalized cluster number for each task epoch, which is the average of the cluster numbers from the five subperiods. A repeated-measures ANOVA showed a significant effect of task epoch for cluster number ( F(2.75,1045.55)=2,795.86,p<0.001, Greenhouse–Geisser correction for sphericity). Post hoc analyses indicated significant differences in the normalized cluster number for each of the transitions in Figure 6*e* (Holm's test, p<0.001 for all the transitions).

### Sliding window analysis of LFP clusters

**Figure 7.**
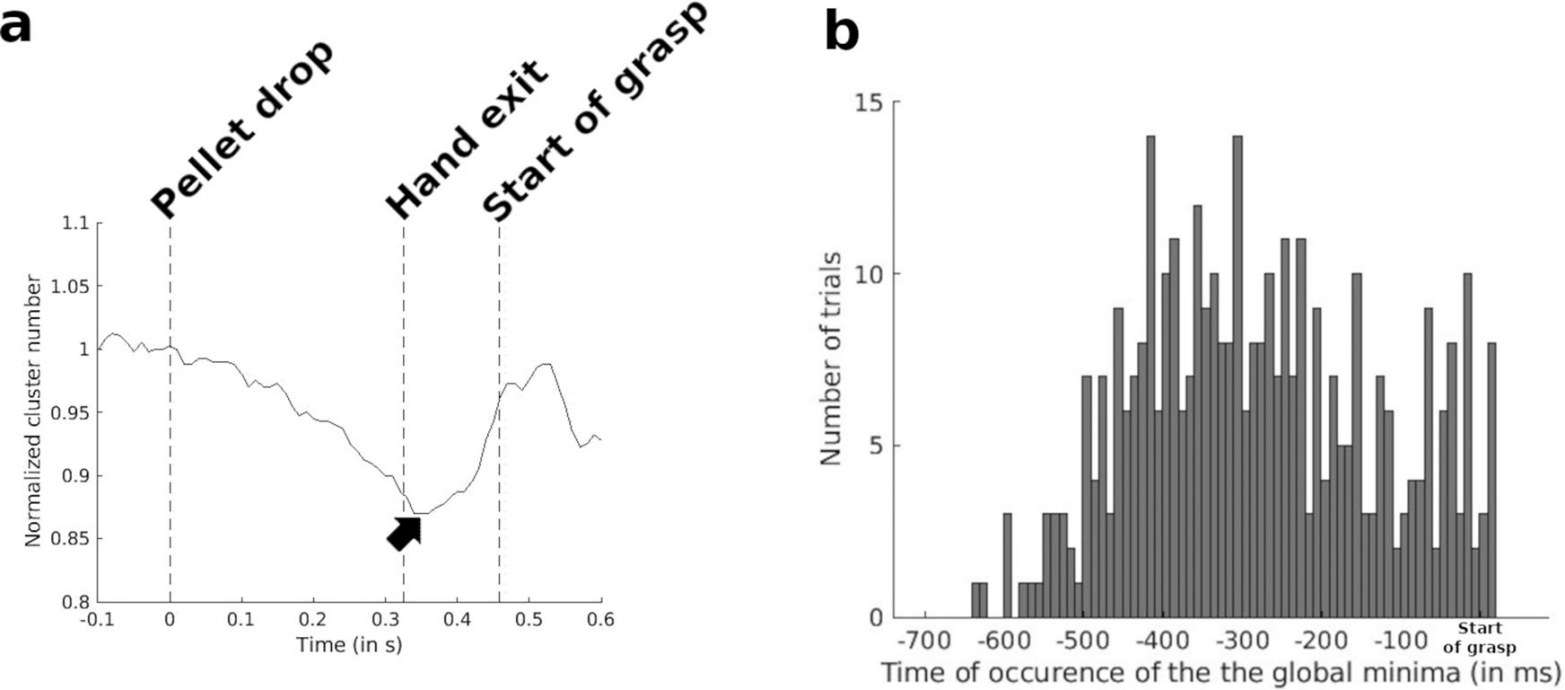
***a***, Smoothed trajectory of continuous estimated cluster number for one trial of a session of Monkey S. The black arrow indicates the global minimum between Pellet drop and Start of grasp. From all recorded trials, a total of eight trials (2.11%) did not show a minimum before Start of grasp. ***b***, Distribution of the number of trials, with time occurrence of the global minimum as a function of time delay from the Start of grasp. All delays are aligned to the Start of grasp as *t* = 0. Negative values would indicate that the normalized cluster number reached a minimum before the Start of grasp.

The section above using coarse subdivisions of our time series showed that mean cluster numbers evolve as a function of task epoch. The next step of our analysis was to examine this feature in more detail using a sliding window analysis. The revelation of salient characteristics in this feature before the start of task epochs could prove promising for their prediction. To this end, the clustering was performed using a sliding window of 100 ms instead of the subperiods used in the previous section. The result of the sliding window analysis for one example trial is presented in Figure 7*a*.

One particular feature of this continuous estimated cluster number was the occurrence in the vast majority of cases, of an inflection in the cluster number curve following pellet drop (Fig. 7*a*). This feature resulted in the existence of a global minimum between Pellet drop and Start of grasp (Fig. 7*a*, black arrow). For 97.89% of all recorded trials, this global minimum was found to occur before the Start of grasp (one-sample *t* test, *p *< 0.001 for all trial conditions). It occurred on average at a distance of 285 ± 8 ms before the start of grasp (Fig. 7*b*). A chi-square test comparing the distribution of Figure 7*b* with that of a uniform distribution showed a significant difference (Pearson's chi-square test, $\chi^{2}(74)=214.31\;,\;p<0.001$). This confirmed the presence of a bias for the time occurrence of this global minimum for certain intervals before the start of grasp.

## Discussion

In this study, we focused on how grouping in LFP activities recorded by multiarray electrodes evolves as a function of a reach-and-grasp task. The analysis was done using correlations and hierarchical clustering. Correlations were high when Reach started and decreased by the time of grasping. The increase in correlations with reach is what would be expected from several previous studies at the behavioral and muscular level. Researchers have demonstrated a quasi-linear relationships between the kinematic angles of the arm during reach (Soechting and Lacquaniti, 1981; Lacquaniti et al., 1986). At the muscular level, indications of correlations during reach have also been demonstrated with the computation of a small number of synergies which are able to reproduce the muscular activation patterns for reaching at different speeds, in different directions and with different loads (d’Avella et al., 2008, 2011; Muceli et al., 2010). Covariations in muscular activations patterns have also been described for finger movements (Weiss and Flanders, 2004; Klein Breteler et al., 2007; d’avella and Lacquaniti, 2013; Geed and van Kan, 2017; Pei et al., 2022). However, correlated activity during grasp can be expected to be lower than what is found during reaching as the fingers have to act in a more individuated manner. Previous research has shown that the number of components required to capture the variance during reach (d’Avella et al., 2008; Muceli et al., 2010) has been generally smaller than those needed for tasks involving the hand (Weiss and Flanders, 2004; Mollazadeh et al., 2014; Takahashi et al., 2017). In keeping with this, the LFPs in our current study showed a decrease in correlation and increase in the cluster number as Grasp began.

Rather than working with mean values and relying on statistical effects, we chose to examine the patterns of correlations and cluster formations separately for each type of reach-and-grasp. This pattern of increasing correlation during Reach followed by a decrease during Grasp was very robust, following the same organization, irrespective of arm used and hand orientation. In future studies, it will be interesting to carry out the same computation for other types of reach-and-grasp tasks to see if it is an invariant LFP characteristic for this type of motor task.

The algorithm chosen to investigate the grouping of the LFP activities in more detail was hierarchical clustering. Compared with PCAs, this method affords much greater ease of interpretation as each electrode belongs to one particular cluster as opposed to PCAs, in which the activity recorded by a given electrode can be distributed across several components. Hierarchical clustering was also chosen to avoid some difficulties found with other clustering algorithms. For example, with *k*-means (Lloyd, 1982) and Gaussian mixture models (Dempster et al., 1977), the number of clusters is determined a priori. One could decide to change cluster numbers if the number originally proposed does not work, but the algorithm begins with a proposed cluster number. Hierarchical clustering does not have this prerequisite. This method also provides a hierarchy of similarity between data points, hence allowing for the progressive quantification of data similarity. The criterion chosen to create clusters was correlation. This was primarily due to our interest in motor control and muscular correlations in movement. Having said that, clustering can also be done using other criteria such as Euclidian distances. We attempted clustering using this criterion and did not obtain similar results (figure not included). This does not of course invalidate our results as different clustering patterns can be obtained when different criteria are applied. We would also argue that correlation is a stronger indicator of signal similarity. Two high-amplitude signals that show closely varying trajectories are likely to show high correlation but be separated by a big Euclidian distance as even small shifts would give rise to high distance values.

Our study joins several others that have found useful indicators concerning movement in LFP signals (Mehring et al., 2003; Scherberger et al., 2005; Spinks et al., 2008; Bansal et al., 2012; Flint et al., 2012; Milekovic et al., 2015; Falaki et al., 2024). Most of these did not investigate the question of correlations between LFP activities. For example, Mehring et al. (2003) used LFPs recorded within the motor cortex to infer which arm had been used as well as to decode hand position and movement velocity. The researchers found improved predictions by adding in the contributions of multiple electrodes but did not explore the question of movement information encoding in the correlations of LFP activities. Concerning movement planning, Scherberger et al. (2005) showed how LFP activity before the start of reaching was different from that before a saccade in the posterior parietal cortex. This study used single electrode LFP recordings. Hence decoding concerning reach and saccades originated in single unit properties—in this case, the LFP amplitudes at specific frequencies. Spinks et al. (2008) demonstrated selectivity in the beta frequencies for six objects in the primary motor and ventral premotor cortex. They found poor correlation in the object tuning between the LFP recordings from M1 and the ventral premotor cortex of the monkey but did not seek to follow the nature of these correlations over the course of reach-and-grasp.

An example of a study focused on the question of correlations during reach-and-grasp is a study by Quarta et al. (2022). These researchers used hierarchical clustering to examine the evolutions of clustering in signals from calcium imaging during a reach-and-grasp task in mice. In agreement with our results, they also found a minimum Ward distance (which translates to a minimum cluster number in our study) right before Reach onset and a maximum at the Start of grasp. In contrast to our study, the Quarta et al. (2022) investigation with calcium imaging was a more coarse-grained study demonstrating how neuronal connectivity related to the reach-and-grasp could involve not only the motor cortex but several non-motor areas such as the visual, somatosensory, and retrosplenial cortex. Our study with >100 electrodes in the motor areas provides a demonstration that a similar organization can be seen at a finer-grained level. Another example is also an investigation by DePass et al. (2022) conducted in the motor cortex. They reported a better accuracy for distinguishing five task epochs during reach-and-grasp by using the LFP spectral power at high frequencies. They reported that correlations between LFPs of the electrodes only provide a poor prediction of task epoch. It should be emphasized that the aim of our current study was not to carry out any decoding but to probe the presence of LFP signal properties pertinent to a key feature in motor control which is that of synergy formation. Indeed as the LFP correlations increased with reach and then decreased again, over the course of Grasp, it is quite possible that a decoder would have trouble distinguishing some phases of movement preparation and grasp using this feature.

Following the observations with mean values of cluster numbers during the subperiods of each epoch, we decided to monitor the evolution of these cluster numbers with a finer probe using a sliding window analysis. Such a procedure could allow us to detect features leading up to key events like the Start of grasp. Figure 7 shows how there is a progressive decrease in cluster number during reach which attains a minimum before a rebound as Grasp approaches. This global minimum could therefore serve as a marker of increased probability of the Start of grasp. Such a marker could be useful as it would indicate the point at which the LFPs have to be analyzed for the type of grasp that was performed. This would provide an easier classification task than one which analyzes the entire time series from the start of Reach. Other studies have reported on LFP features which can serve as temporal markers of task epochs. Both Scherberger et al. (2005) and Hwang and Andersen (2009) report that LFP power in the posterior parietal cortex increases for the low-frequency bands and decrease for the high-frequency bands during the phase between planning and execution for reaching. Decoding of trial phase in the motor cortex was also done by Falaki et al. (2024) and Orellana et al. (2024). Compared with these previous studies, ours is a study which attempts to predict an oncoming event rather than detecting events which have already started. Another contrast with previous studies is the use of cluster number rather than spectral amplitude. However, many improvements will have to be put in place before this global minimum in cluster number can be used as an online detector of the Start of grasp. For one, studies using various widths of the sliding window will have to be conducted to find the window of optimal size. Second, trials of a longer duration would have to be conducted to further verify that the global minimum does indeed occur close to Grasp onset. Finally, correlation is computationally expensive, and while theoretical tools can indicate the presence of promising tools, further improvements in computational speed would have to be made before the practical use of such a minimum point in cluster number. Its occurrence in almost every trial, at a mean distance of ∼285 ms from the Start of grasp, shows that it may be used as a marker indicating an increased probability of Start of grasp.

Although Figure 4*a*,*b* demonstrated the presence of the correlation transitions as a function of task epochs at all frequencies, we chose to focus our attention on the delta band as it showed the highest changes in amplitudes in the trial spectrograms. The amplitude increases in the gamma band of our study were not as high. This is in contrast to many researchers who have instead observed higher changes in the gamma band during the start of grasp (Menzer et al., 2014; Milekovic et al., 2015; Perel et al., 2015; Waldert et al., 2015). However, this clear transition of spectral power in the gamma band is not present for all research on LFPs and hand movement. The Witham and Baker study (Witham and Baker, 2012) shows poor increase in the gamma band during finger movements. Spinks et al. (2008) demonstrated greater selectivity for some grasp types in the beta band than at higher frequencies. A closer look at papers like Waldert et al. (2015) also shows that the increase in the gamma band is not consistent and varies with the monkey and the brain area being examined. The Falaki et al. (2024) study on reach and grasp in the motor cortex shows significantly higher spectral amplitude in the delta band than in the gamma band during movement.

Since in many cases, the increase in the amplitude of the delta band occurs before the start of movement, there have been suggestions that it is more associated with attentional mechanisms corresponding to stimulus saliency (Saleh et al., 2010; Popovych et al., 2016) rather than directly with the movement itself. The research from several groups would belie this claim. First of all, several researchers have found detailed information concerning the type and kinetics of reach and grasp in low-frequency bands. Examples of this are Milekovic et al. (2015), Bansal et al. (2012), Orellana et al. (2024), and Falaki et al. (2024). Milekovic points out that the low and high frequencies provide similar information concerning the task. The involvement of the LFP low-frequency components in Milekovic study is especially convincing when the delta band is used to provide a continuous decoding of grip parameters during task execution. Second, the decrease of delta power before the start of movement need not indicate an association with attentional mechanisms as opposed to a direct relationship with muscular activity. The anticipative postural adaptations (APAs) involving upper and lower limb movements are seen in EMG modulation before the start of visible movement. While most of the work on APAs has focused on postural adjustments in the lower limbs (Massion, 1992; Stapley et al., 1998; Santos et al., 2010), many researchers have also demonstrated their presence in the upper limbs. A review article on this was written by Cavallari et al. (2016).

In our study the computation of correlations and clusters was carried out over all the implanted arrays of the motor cortex. This included the primary motor cortex for one hemisphere as well as the dorsal and ventral premotor cortexes of both hemispheres. This was in keeping with several human and animal studies that would seem to indicate overlapping activations of all these areas during the task. For example, there are observation from human studies of a close coordination between reach and grasp in which the two take place at the same time and influence each other (Jeannerod, 1984; Wallace et al., 1990). As a result of such observations at the behavioral level, Takahashi et al. (2017) undertook an investigation in which they decided to investigate the classical separation of roles between the dorsal and ventral premotor cortex. They found that neurons in both areas play a role in reach as well as grasp. Neural activity even in the case of unimanual movements is known to involve ipsilateral circuits as well (Moreau-Debord et al., 2021).

In conclusion, our study shows that alterations in correlations and cluster formation of LFP activity measured using multiarray electrodes in the motor cortex are consistent with what would be expected from several lines of research at the behavioral level of the reach-and-grasp task. Hierarchical cluster analyses of LFP spectral power in the delta band showed a consistent decrease in normalized cluster number as Reach started and increased during Grasp. Since LFP recordings are easier and more robust to obtain than neuronal recordings, this shows a promise for the monitoring of motor control of muscular disorders involving abnormal synergistic activities.

## Synthesis

Reviewing Editor: David Franklin, Technische Universitat Munchen Decisions are customarily a result of the Reviewing Editor and the peer reviewers coming together and discussing their recommendations until a consensus is reached. When revisions are invited, a fact-based synthesis statement explaining their decision and outlining what is needed to prepare a revision will be listed below. The following reviewer(s) agreed to reveal their identity: NONE. Note: If this manuscript was transferred from JNeurosci and a decision was made to accept the manuscript without peer review, a brief statement to this effect will instead be what is listed below.

Your manuscript has now been reviewed by two experts in the field. They both think that this work is important as there is little research investigating correlations in the local field potential recordings during reach and grasp tasks. However, we all have some concerns with the current manuscript. Specifically, we are concerned with the lack of specific details in the methods and the manner in which the data and algorithm is selected, the specific interpretation of the study, and the window size over which the algorithm is used for prediction. We are concerned as the window size effects the practicality of using the technique for prediction. We encourage you to revise the manuscript, taking these and the following detailed descriptions of the specific issues from the reviewers into account.

General Comments from the reviewers.

The authors investigated modifications in the correlation and clustering of Local field Potential (LFP) recordings during reach-and-grasp tasks in non-human primates. Regions of interest are M1, PMd, PMv. The recordings were made while monkeys reached food pellets with different orientation of the hand. The authors analyze the data through correlation analysis and hierarchical clustering on the LFP recordings. The authors claim that normalized cluster number in the delta band decreases as reach starts and the number increases during grasp.

Major critical points:

The authors suggest an application of the prediction of the grasp-start timing based on the cluster number. However, I have serious doubts about its practical applicability.

First, it is based on the delta-band LFP amplitude, and it is obtained with a taper method using 400 ms sliding window. This means that one can obtain an estimate of the LFP amplitude at time t only after observing the original data until t + 200 ms. Since the authors report that the typical latency to the grasp-start from the predicting event (i.e., the cluster number reaches 90% of the baseline) is 80 ms, when this event is detected (using the data 200 ms ahead), the grasp-start has already occurred. For a prediction to be practically useful, it should be based only on the data in the past. The authors should take this aspect into consideration and revise the manuscript in the parts related to the prediction.

- Experimental model and surgery: "Here, we present results from two sessions recorded in monkey M and two sessions in monkey S during which neuronal recordings were obtained from most of the implanted brain areas." Monkey 1 has 370 electrodes implanted while monkey 2 has 448. Their acquisition board can record a maximum number of 256 simultaneous channels so different subset of cells are selected. They fail to mention how they choose those channels. They had a preliminary analysis? They are selected randomly? Why not compute the analysis with different neurons selected to show robustness in choosing such channels?

- Hierarchical clustering: 'This distance criterion was set during the Observation phase'. Since the authors are studying reach and grasp it is not clear why they set the distance criterion during the Observation phase and not during the reach phase, which present very different spectral profiles.

- Hierarchical clustering: 'In our case, the distance measure used to evaluate the separation between two LFP electrodes was correlation'. It might be useful to explain why the author chose correlation as metric of distance instead of Euclidean distance and/or cosine similarity. This is an important clarification because hierarchical clustering heavily relies on starting points and metric utilized. Given this sensitivity of the analysis implemented it would be nice to show results with different metrics to provide the reader with information about the stability of the analysis implemented.

- Results: 'Others have also reported the link between activity in the delta band of the motor cortex and arm movement (Milekovic et al., 2015; Rickert et al., 2005; Waldert et al., 2015; Zhuang et al., 2010).'

o Milekovic et al. 2015: it is shown that all frequencies 0.3-7Hz, 10-40Hz and 80-250Hz present increased amplitude, not just the delta. Furthermore, they show how lower and higher frequencies provide similar decoding accuracies so there must be at least a comparable role for lower and higher frequencies in term of importance.

o Waldert et al. 2015: it is shown that over M1 beta and gamma bands present the most precise increase in power for movements, while delta power increases before movement onset, indicating its relevance to attentional mechanisms rather than movement per se (as shown also in Saleh et al. 2010).

o Zhuang et al. 2015: it is shown that the best performing substrate to decode movement related parameters are the gamma bands, while delta activity is not as predictive as other bands.

Taken together these references rather than indicating a direct link between delta band and arm movement, they suggest a second order connection between delta bands and arm movements, which is mediated by the role of delta bands on attentional mechanisms which in turn exert a direct effect on arm movement.

- Results: 'The analyses of the study were focused on the delta band as we found the largest modulations of spectral power in this band following pellet drop'. Pellet drop is an example of behaviorally relevant cue, and it has been shown in literature that delta band is very much linked to these types of stimuli rather than movement per se (Saleh at al.2015). To resolve this issue the message of the paper should be modified i.e. instead of delta bands are modulated by movement, delta bands are modulated by saliency, which in turn influences movement.

Minor:

- Introduction: the authors claim that LFP are less subject to electrode position compared to spikes, but they fail to mention that this signal is also more noisy and more prone to artifacts.

- Fig.1: 'More details on the manner in which each epoch was defined can be found in the Methods section.' It would be clearer to specify in which subsection they deal with epoch definition.

- Table 1: they present the number of trial conditions however, they range from 26 to 20, which is a 30% variability. This huge variability might impact the results, it might be nice to choose the condition with the least trials and discard all the trials in excess for the other conditions.

Fig.2: There is a non-negligible amount of literature that found sharp increases in PSD in the gamma bands when movement starts, i.e. Waldert et al. 2015 which they cite. Fig.2 does not show this sharp increase in the gamma bands and in the text it is not explained why their results are not in accordance with the majority of literature. Furthermore, it is not specified from which animal the PSD has been computed for this figure. It might be useful to check literature on the role of delta bands on attentional mechanisms, rather than arm movements.

- Preprocessing: the authors use a single tapering method with 10ms of padding to compute spectrograms. No information is provided regarding the type of window used. However, the authors cite Waldert et al. 2015 which uses a zero padding and Menzer et al. 2014 which uses 5 different tapers, so the methods are not comparable between this work and relative citations. The type of window utilized must be specified.

- Correlations: 'average correlations was obtained from the absolute values of the pairwise correlations'. So if there was a negative correlation in any pairwise correlation they just switch the sign? This is not clear.

- Fig. 3: it is not specified from which animal they computed the hierarchical clustering.

- Fig.4-5: 4a and 4b show results for monkey S while fig.5 shows results from monkey M, this is not consistent.

- Prediction: 'One particular feature of the continuous estimated cluster number was the occurrence in the vast majority of cases, of an inflection in the cluster number curve following pellet drop'. Again, this is pointing out to the relevance of delta bands for behaviorally relevant cues, not for movement.

- Prediction: the authors claim that their sliding window approach might be suitable for online analysis. This might present two main issues: 1 all the analysis performed are computationally expensive and 2 the window to predict the grasp is 80 ms wide, which is not precise enough for efficient online classification. I will avoid suggesting that this work might be deployed as an online decoder.

- Figure 7a: it would be nice to show confidence interval as std.

- Discussion: to substantiate their claims they show how in literature muscular activity follows certain correlation of activity, which is not relevant to the main objective of the paper since its focus is on neural coherence, not muscular, which might follow the same pattern, but it is not always the case.

- Discussion: the authors claim that hierarchical clustering is better suited to this type of analysis compared to k-means clustering because k-means has a fixed number of clusters, which is not how k-means works. They also fail to mention that hierarchical clustering is much more sensitive to starting point and metric utilized compared to k-means. So, it would be appreciable to further clarify why hierarchical clustering has been chosen over other types of analysis.

- Discussion: the authors claim to extend the results of Spinks et al. 2008, but in that work the authors 1 did not find any sign of delta band for movement and 2 they find increased power in the beta regime, which is completely neglected by this work. I would remove the part linking the current work to the work of Spinks et al. 2008.

- Discussion: 'Since LFP recordings are easier and more robust to obtain than neuronal recordings' This is simply not accurate.

- In Fig. 7b, the timing of "start of grasp" in relation to the peak of the histogram is not clear.

Please indicate this timing clearly, probably with a salient vertical line.

- The caption to Fig. 7 tells that the curve in the plot is smoothed.

How was it smoothed?

And is the prediction based on the smoothed curve, or the original, un-smoothed curve?

There are some more missing pieces of information about the methodological details for the prediction analysis (such as the window step for the sliding window used to continuously estimate the cluster number). Those details should be described in the methods section.

And there is yet another concern, which is not specific to the prediction part: in the "Correlation" paragraph of the methods section, the authors wrote "the final correlation values analyzed were an average of correlations across the delta band", but "the delta band" is not explicitly and clearly defined in the manuscript. Please clearly define the delta band used in the present study.

## Author Response

Response to reviewers Reviewer comment 1 1. The authors suggest an application of the prediction of the grasp-start timing based on the cluster number. However, I have serious doubts about its practical applicability. First, it is based on the delta-band LFP amplitude, and it is obtained with a taper method using 400 ms sliding window. This means that one can obtain an estimate of the LFP amplitude at time t only after observing the original data until t + 200 ms. Since the authors report that the typical latency to the grasp-start from the predicting event (i.e., the cluster number reaches 90% of the baseline) is 80 ms, when this event is detected (using the data 200 ms ahead), the grasp-start has already occurred. For a prediction to be practically useful, it should be based only on the data in the past. The authors should take this aspect into consideration and revise the manuscript in the parts related to the prediction.

Answer to Comment 1 The reviewers are right in pointing out that our claims on prediction need to be improved as the delta band was done using a taper window of 400ms. In order to bring us to time points which take this into account, we have moved back to a point in the LFP cluster number time series which we were considering originally - the global minimum in the cluster number curve between Pellet Drop and Start of Grasp. This minimum occurs at a mean value of 258{plus minus}8 ms before the Start of Grasp. This whole section has been re-done. The title of the section which was 'Prediction' is now entitled 'Sliding Window Analysis of LFP Clustering'.

We have also added more nuanced sentences on the contribution of the global minimum to detecting the onset of Grasp with the following sentence in the abstract. Rather than reporting that this would allow us to predict the imminent onset of Start of Grasp, we have instead reported that it indicates an increased probability of the Start of Grasp.

A sliding window computation of the number of clusters was performed to probe the capacities of these LFP clusters for detecting upcoming task events. For a very high percentage of trials (95.3%), there was a downturn in cluster number following the Pellet Drop (GO signal) which reached a minimum preceding the Start of grasp, hence indicating that cluster analyses of LFP signals could add to signaling the increased probability of the Start of grasp.

The Discussion section on the global minimum in cluster number has also been modified to compare our study on Task Epochs with those of previous authors. Once again, we have tried to be careful in nuancing our claims on the prediction of imminent Start of Grasp, to one of indicating increased probability of Start of Grasp. We have also included in this section all the limits and improvements that would be required for such a computation to have practical application.

Following the observations with mean values of cluster numbers during the sub periods of each epoch, we decided to monitor the evolution of these cluster numbers with a finer probe using a sliding window analysis. Such a procedure could allow us to detect features leading up to key events like the Start of Grasp. Figure 7 shows how there is a progressive decrease in cluster number during reach which attains a minimum before a rebound as Grasp approaches. This local minimum could therefore serve as a marker of increased probability of the Start of grasp. Such a marker could be useful as it would indicate the point at which the LFPs have to be analyzed for the type of grasp that was performed. This would provide an easier classification task than one which analyzes the entire time series from the start of Reach. Other studies have reported on LFP features which can serve as temporal markers of task epochs. Both Scherberger et al. (Scherberger et al., 2005) and Hwang &Andersen (Hwang &Andersen, 2009) report that LFP power in the posterior parietal cortex increases for the low frequency bands and decrease for the high frequency bands during the phase between planning and execution for reaching. Decoding of trial phase in the motor cortex was also done by Falaki (Falaki et al, 2024) and Orellana (Orellana et al, 2024). Compared to these previous studies, ours is a study which attempts to predict an oncoming event rather than detecting events which have already started. Another contrast with previous studies is the use of cluster number rather than spectral amplitude. However, many improvements will have to be put in place before this local minimum in cluster number can be used an online detector of the Start of Grasp. For one, studies using various widths of the sliding window will have to be conducted to find the window of optimal size. Second, trials of a longer duration would have to be conducted to further verify that the global minimum does indeed occur close to Grasp onset. Finally, correlation is computationally expensive and while theoretical tools can indicate the presence of promising tools, further improvements in computational speed would have to be made before the practical use of such a minimum point in cluster number. Its occurrence in almost every trial, at approximately 260 ms from the Start of Grasp shows that it may be used as a marker indicating an increased probability of Start of Grasp.

Reviewer comment 2 2. Experimental model and surgery: "Here, we present results from two sessions recorded in monkey M and two sessions in monkey S during which neuronal recordings were obtained from most of the implanted brain areas." Monkey 1 has 370 electrodes implanted while monkey 2 has 448. Their acquisition board can record a maximum number of 256 simultaneous channels so different subset of cells are selected. They fail to mention how they choose those channels. They had a preliminary analysis? They are selected randomly? Why not compute the analysis with different neurons selected to show robustness in choosing such channels? Answer to comment 2 The electrodes were not individually chosen. Instead, connectors each collecting data from 32 electrodes were picked for each session. They were picked to collect data from the various areas of interest in the motor cortex. In this case, M1, pMd and pMv for all the sessions. The electrode sets mentioned in the paper are therefore never the same. Therefore, the suggestion of the reviewer has already been implemented. The results appear quite robust - four different sets of electrodes show the clustering patterns that we present in the paper.

Reviewer comment 3 3. Hierarchical clustering: 'This distance criterion was set during the Observation phase'. Since the authors are studying reach and grasp it is not clear why they set the distance criterion during the Observation phase and not during the reach phase, which present very different spectral profiles.

Answer to comment 3 The distance criterion was set during the observation phase as we wanted to follow the evolution in correlations and cluster formation from the observation period to reach, and then from reach to grasp. In other words, we were not only interested in correlation transitions from reach to grasp, but also from rest to the start of reach. Both transitions are important in the field of motor control.

Concerning the comment about the differences in spectral profile of reach, Figure 4a, shows that the correlations showed a similar pattern of evolution at all frequencies. Since the criterion used for cluster formation was correlation, it is likely that we would have observed the same pattern of cluster formation even if we started in the reach phase with a different spectral profile. In response to the reviewers comments, we have also added figure 4b showing that the transitions at all frequencies were also observed for a second monkey.

Reviewer comment 4 4. - Hierarchical clustering: 'In our case, the distance measure used to evaluate the separation between two LFP electrodes was correlation'. It might be useful to explain why the author chose correlation as metric of distance instead of Euclidean distance and/or cosine similarity. This is an important clarification because hierarchical clustering heavily relies on starting points and metric utilized. Given this sensitivity of the analysis implemented it would be nice to show results with different metrics to provide the reader with information about the stability of the analysis implemented.

Answer to reviewer comment 4 We do not think that differences in result using a different clustering criterion would have invalidated our results. Different clusters could be formed when using different criteria. The next step then would be the requirement of having an agent or agents (for want of a better word) capable of tapping into these different sources of information (eg neural nets with different hidden layer filters). Having said this, we will explain below why we favoured the use of correlations.

Correlation was chosen as a measure of clustering for several reasons a. As explained in the introduction of the paper, research in the field of motor control has clearly demonstrated changes in the correlation of muscular activity during movement. Going on this cue, we started out by examining the changes in the correlation of LFP spectral power with task epochs. The simple correlation analysis of figure 4 showed that this correlation undergoes robust changes as a function of task epoch. b. It seemed like a more robust measure. The Euclidian distance would be subject to factors like amplitude. Two LFP signals with a similar form but high amplitude would therefore show greater Euclidian distances from each other, than two low amplitude LFP signals even if they have a similar waveform.

We have added this explanation to the Discussion section.

The criterion chosen to create clusters, was correlation. This was primarily due to our interest in motor control and muscular correlations in movement. Having said that, clustering can also be done using other criteria such as Euclidian distances. We attempted clustering using this criterion and did not obtain similar results (figure not included). This does not of course invalidate our results as different clustering patterns can be obtained when different criteria are applied. We would also argue that correlation is a stronger indicator of signal similarity. Two high amplitude signals that show closely varying trajectories are likely to show high correlation, but be separated by a big Euclidian distance as even small shifts would give rise to high distance values.

Reviewer comment 5 5. - Results: 'Others have also reported the link between activity in the delta band of the motor cortex and arm movement (Milekovic et al., 2015; Rickert et al., 2005; Waldert et al., 2015; Zhuang et al., 2010).'o Milekovic et al. 2015: it is shown that all frequencies 0.3-7Hz, 10-40Hz and 80-250Hz present increased amplitude, not just the delta. Furthermore, they show how lower and higher frequencies provide similar decoding accuracies so there must be at least a comparable role for lower and higher frequencies in term of importance. o Waldert et al. 2015: it is shown that over M1 beta and gamma bands present the most precise increase in power for movements, while delta power increases before movement onset, indicating its relevance to attentional mechanisms rather than movement per se (as shown also in Saleh et al.2010). o Zhuang et al. 2015: it is shown that the best performing substrate to decode movement related parameters are the gamma bands, while delta activity is not as predictive as other bands. Taken together these references rather than indicating a direct link between delta band and arm movement, they suggest a second order connection between delta bands and arm movements, which is mediated by the role of delta bands on attentional mechanisms which in turn exert a direct effect on arm movement.

Results: 'The analyses of the study were focused on the delta band as we found the largest modulations of spectral power in this band following pellet drop'. Pellet drop is an example of behaviorally relevant cue, and it has been shown in literature that delta band is very much linked to these types of stimuli rather than movement per se (Saleh at al.2015). To resolve this issue the message of the paper should be modified i.e. instead of delta bands are modulated by movement, delta bands are modulated by saliency, which in turn influences movement.

Reply to reviewer comment 5 The reviewer is right in pointing out that several researchers have demonstrated the presence of decoding information in the spectral power at several frequencies. However, there are several things to say about this: a) The main gist of the paper is not to provide decoding information, but to point out the presence of characteristics in LFP activity which correspond to a cardinal feature of motor control, namely, synergy formation in movement. The introduction of the paper has laid out this focus. Synergy formation has clearly been seen at the muscular level and a few subsequent studies using neuronal recordings and fMRi have pointed to the presence of low dimensional activities in the brain during movement. Our goal was to probe the presence of such correlations in LFP recordings. Of course, many more studies will have to be conducted to demonstrate a causal link between the observation at the neuronal and behavioural level. b) Figure 4a of the previous version of the paper demonstrated that indeed, the transitions in correlations as a function of task epochs, could be seen at the lower and higher frequencies. In order to emphasize this point and address the concerns of the reviewer, we have now added an additional figure.

We have added a new figure 4b, which displays the transitions from one trial at all frequencies for the second monkey M. c) The reviewer is of the point of view that the activity of the delta band is modulated by saliency rather than movement per se. After a further survey of the literature, we found that the opinion on this is not homogenous. We would respond in the following manner to this opinion i. Several researchers have found detailed information concerning the type and kinetics of reach and grasp in low frequency bands. Examples of this are Milekovic et al (2015), Bansal et al (2011), Orrelana et al (2024) and Falaki (2024). Milekovic points out that the low and high frequencies provide similar information concerning the task. The involvement of the LFP low frequency components is especially convincing when they are used to provide a continuous decoding of grip parameters during task execution. ii. The decrease of delta power before the start of movement need not indicate an association with attentional mechanisms as opposed to a direct relationship with muscular activity. The anticipative postural adaptations (APA) involving upper and lower limb movements are seen in EMG modulations before the start of visible movement. While most of the work on APA's has focused on postural adjustments in the lower limbs (Massion 1992; Stapley et al, 1998; Santos et al, 2010) many researchers have also demonstrated their presence in the upper limbs. A review article on this was written by Cavallari et al, 2016.

To take into account the comments of the reviewer on this point, we have added additional paragraphs to the Discussion, which address the question of low amplitudes in the gamma range as well as the question of the significance of activity in the delta band. This paragraph can be seen below.

Since in many cases, the increase in the amplitude of the delta band occurs before the start of movement, there have been suggestions that it is more associated with attentional mechanisms corresponding to stimulus saliency (Popovych et al., 2016; Saleh et al., 2010) rather than directly with the movement itself. The research from several groups would belie this claim. First of all, several researchers have found detailed information concerning the type and kinetics of reach and grasp in low frequency bands. Examples of this are Milekovic et al (2015), Bansal et al (2011), Orrelana et al (2024) and Falaki et al (2024). Milekovic points out that the low and high frequencies provide similar information concerning the task. The involvement of the LFP low frequency components in Milekovic study is especially convincing when the delta band is used to provide a continuous decoding of grip parameters during task execution. Second, the decrease of delta power before the start of movement need not indicate an association with attentional mechanisms as opposed to a direct relationship with muscular activity. The anticipative postural adaptations (APA) involving upper and lower limb movements are seen in EMG modulations before the start of visible movement. While most of the work on APA's has focused on postural adjustments in the lower limbs (Massion, 1992; Santos et al., 2010; Stapley et al., 1998, Thomas et al, 2012) many researchers have also demonstrated their presence in the upper limbs. A review article on this was written by Cavallari et al, 2016.

Reviewer comment 6 (Minor comment) 6. - Introduction: the authors claim that LFP are less subject to electrode position compared to spikes, but they fail to mention that this signal is also more noisy and more prone to artifacts.

Answer to reviewer comment 6 There is quite a volume of studies demonstrating reliable decoding of motor activity using LFPs, including continuous decoding. We felt therefore that a more serious criticism of the technique is one concerning the difficulties of interpretation concerning underlying neural mechanisms (Herreras, 2016). We have therefore added this to the Introduction As in the case of the electroencephalogram, it is difficult to draw conclusions concerning their significance and origins, hence limiting their usefulness for understanding the underlying mechanisms (Herreras et al, 2016) Reviewer comment 7 (Minor comment) 7. - Fig.1: 'More details on the manner in which each epoch was defined can be found in the Methods section.' It would be clearer to specify in which subsection they deal with epoch definition.

Answer to comment 7 This improvement has been added to the figure caption of Figure 1 'More details on the manner in which each epoch was defined can be found in the subsection entitled 'Behavioral Task' of the Methods section Reviewer comment 8 (Minor comment) 8. - Table 1: they present the number of trial conditions however, they range from 26 to 20, which is a 30% variability. This huge variability might impact the results, it might be nice to choose the condition with the least trials and discard all the trials in excess for the other conditions.

Response to Reviewer comment 8 As the correlation is a measure which compares electrode activity within each trial, it is very unlikely that differences in trial number would make any difference, especially with trial numbers which are not that small (all sessions had 20 or more trials) and a large number of electrode pairs for each trial (min 131 electrodes).

Nevertheless to confirm this supposition, we have checked if there are statistically significant differences in correlation arising from the number of trials. The biggest difference in trial number is in S19 where there are 25 trials for a right hand horizontal grasp, but only 20 trials for the right hand vertical grasp. A t-test was conducted to check if there was a statistically significant difference in the average correlation during reach between 20 right hand vertical trials but a varying number of right hand horizontal trials going from 15 to 25. A table of the p values from these comparisons has been inserted into the paper (Table 2). None of the p values were significant hence indicating that such differences in trial numbers do not produce differences in correlation values.

Reviewer comment 9 (Minor comment) 9. Fig.2: There is a non-negligible amount of literature that found sharp increases in PSD in the gamma bands when movement starts, i.e. Waldert et al. 2015 which they cite. Fig.2 does not show this sharp increase in the gamma bands and in the text it is not explained why their results are not in accordance with the majority of literature. Furthermore, it is not specified from which animal the PSD has been computed for this figure. It might be useful to check literature on the role of delta bands on attentional mechanisms, rather than arm movements.

Response to Reviewer comment 9 (Minor comment) Concerning the increase in the gamma band, the following paragraph has been added in the Discussion section Although figures 4a and b demonstrated the presence of the correlation transitions as a function of task epochs at all frequencies, we chose to focus our attention on the delta band as it showed the highest changes in amplitudes in the trial spectrograms. The amplitude increases in the gamma band of our study were not as high. This is in contrast to many researchers who have instead observed higher changes in the gamma band during the start of grasp (Perel et al, 2015, Menzer et al, 2014, Waldert et al 2015, Milekovic et al, 2015). However, this clear transition of spectral power in the gamma band is not present for all research on LFPs and hand movement. The Witham and Baker study (2012) show poor increase in the gamma band during finger movements. Spinks et al (2008) demonstrated greater selectivity for some grasp types in the beta band than at higher frequencies. A closer look at papers like Waldert et al (2015) also show that the increase in the gamma band is not consistent and varies with the monkey and the brain area being examined. The Falaki et al (2024) study on reach and grasp in the motor cortex shows significantly higher spectral amplitude in the delta band than in the gamma band during movement.

Concerning whether or not the delta band is more a signature of attentional mechanisms rather than movement per se, the following paragraph has been added Since in many cases, the increase in the amplitude of the delta band occurs before the start of movement, there have been suggestions that it is more associated with attentional mechanisms associated with stimulus saliency (Popovych et al., 2016; Saleh et al., 2010), rather than the directly with the movement itself. The research from several groups would belie this claim. First of all, several researchers have found detailed information concerning the type and kinetics of reach and grasp in low frequency bands. Examples of this are Milekovic et al (2015), Bansal et al (2011) and Orrelana et al (2024). Milekovic points out that the low and high frequencies provide similar information concerning the task. The involvement of the LFP low frequency components in this study is especially convincing when they are used to provide a continuous decoding of grip parameters during task execution. Second, the decrease of delta power before the start of movement need not indicate an association with attentional mechanisms as opposed to a direct relationship with muscular activity. The anticipative postural adaptations (APA) involving upper and lower limb movements are seen in EMG modulations before the start of visible movement. While most of the work on APA's has focused on postural adjustments in the lower limbs (Massion 1992; Stapley et al, 1998; Santos et al, 2010) many researchers have also demonstrated their presence in the upper limbs. A review article on this was written by Cavallari et al, 2016.

Reviewer comment 10 (Minor comment) 10. - Preprocessing: the authors use a single tapering method with 10ms of padding to compute spectrograms. No information is provided regarding the type of window used. However, the authors cite Waldert et al. 2015 which uses a zero padding and Menzer et al. 2014 which uses 5 different tapers, so the methods are not comparable between this work and relative citations. The type of window utilized must be specified.

Reply to Reviewer comment 10 (Minor comment) This information has been included in the Methods section.

The general steps for computing the spectrogram were done following Waldert et al (2015) and Menzer et al (2014). These steps were removing noise from signals, selecting appropriate channels and trials, computing the spectrograms, and finally, normalizing the spectrogram for each trial and frequency using a baseline from the observation phase. More specifically, we used a single taper method with a 400ms sliding Hann window and 10ms time steps.

Reviewer comment 11 (Minor comment) 11. - Correlations: 'average correlations was obtained from the absolute values of the pairwise correlations'. So if there was a negative correlation in any pairwise correlation they just switch the sign? This is not clear.

Reply to Reviewer comment 11 (Minor comment) Yes indeed, negative correlations were turned to positive values. This has been made clearer in the paper. All analyses were done with the absolute values of correlation.

Reviewer comment 12 (Minor comment) 12. - Fig. 3: it is not specified from which animal they computed the hierarchical clustering.

This has been corrected and we have added information on the animal and session in the figure caption.

Reviewer comment 13 (Minor comment) 13. - Fig.4-5: 4a and 4b show results for monkey S while fig.5 shows results from monkey M, this is not consistent.

Reply to Reviewer comment 13 (Minor comment) We are a little confused by this comment. Going through several LFP papers we see that authors do not constrain themselves to using the same monkey for all figures. Figure 4d of the paper contains data from both monkeys. In addition, we have now included a correlation heatmap for a single trial of monkey M to figure 4 (figure 4b).

Reviewer comment 14 (Minor comment) 14. - Prediction: 'One particular feature of the continuous estimated cluster number was the occurrence in the vast majority of cases, of an inflection in the cluster number curve following pellet drop'. Again, this is pointing out to the relevance of delta bands for behaviorally relevant cues, not for movement.

Reply to Reviewer comment 14 (Minor comment) The question of whether the alterations in the delta band are related to signal saliency, or correspond directly to the motor command itself has already been addressed in point 5 of this reply to the reviewer. We have added this paragraph to the Discussion section Since in many cases, the increase in the amplitude of the delta band occurs before the start of movement, there have been suggestions that it is more associated with attentional mechanisms corresponding to stimulus saliency (Popovych et al., 2016; Saleh et al., 2010) rather than directly with the movement itself. The research from several groups would belie this claim. First of all, several researchers have found detailed information concerning the type and kinetics of reach and grasp in low frequency bands. Examples of this are Milekovic et al (2015), Bansal et al (2011), Orrelana et al (2024) and Falaki et al (2024). Milekovic points out that the low and high frequencies provide similar information concerning the task. The involvement of the LFP low frequency components in Milekovic study is especially convincing when the delta band is used to provide a continuous decoding of grip parameters during task execution. Second, the decrease of delta power before the start of movement need not indicate an association with attentional mechanisms as opposed to a direct relationship with muscular activity. The anticipative postural adaptations (APA) involving upper and lower limb movements are seen in EMG modulations before the start of visible movement. While most of the work on APA's has focused on postural adjustments in the lower limbs (Massion, 1992; Santos et al., 2010; Stapley et al., 1998, Tolambiya et al, 2012) many researchers have also demonstrated their presence in the upper limbs. A review article on this was written by Cavallari et al, 2016.

Reviewer comment 15 (Minor comment) 15. - Prediction: the authors claim that their sliding window approach might be suitable for online analysis. This might present two main issues: 1 all the analysis performed are computationally expensive and 2 the window to predict the grasp is 80 ms wide, which is not precise enough for efficient online classification. I will avoid suggesting that this work might be deployed as an online decoder.

Reply to Reviewer comment 15 (Minor comment) This comment has been addressed in the answer to reviewer comment 1.

Reviewer comment 16 (Minor comment) 16. - Figure 7a: it would be nice to show confidence interval as std.

Reply to reviewer comment 16 (Minor comment) We beg to differ on this point. The time base on the trials are not normalized in our study and overlapping several trials of different lengths for this figure will only lead to a loss of the information that we seek to convey ie To show the readers what we mean by the global minimum in the normalized cluster number between Pellet Drop and Start of Grasp.

Reviewer comment 17 17. - Discussion: to substantiate their claims they show how in literature muscular activity follows certain correlation of activity, which is not relevant to the main objective of the paper since its focus is on neural coherence, not muscular, which might follow the same pattern, but it is not always the case.

The discussion concerning muscular correlations, follow Reply to Reviewer comment 17 The paragraph on muscular contractions and correlations in the Discussion section is important. It was not done to substantiate our findings on LFP correlations. Instead, it was done to re-confirm what had been the original intention of the study. The Introduction describes the importance of muscular correlations and synergy in movement. Since this is important in motor control, we explain that we might be able to find it in LFPs. Since we do find correlations in the Results section, we then go on in the Discussion section to comment on the fact that the LFP correlations follow what has been observed in the field of motor control and muscular correlations.

Reviewer comment 18 18. - Discussion: the authors claim that hierarchical clustering is better suited to this type of analysis compared to k-means clustering because k-means has a fixed number of clusters, which is not how k-means works. They also fail to mention that hierarchical clustering is much more sensitive to starting point and metric utilized compared to k-means. So, it would be appreciable to further clarify why hierarchical clustering has been chosen over other types of analysis.

Reply to reviewer comment 18 We were not perhaps clear enough. In K means we have to assign a number of clusters. Of course if the assigned number does not perform the job well, one could change the cluster number. This is not the way in which hierarchical clustering works. There is no pre-determination of cluster number. We have tried to make this point clearer in the Discussion section with the following sentences.

For example, with k-means (Lloyd, 1982) and k-nearest neighbors (Fix &Hodges, 1989) the number of clusters is determined a priori. One could decide to change cluster numbers if the number originally proposed does not work, but the algorithm begins with a proposed cluster number.

We have addressed the point concerning the distance criterion at Review point number 4.

The criterion chosen to create clusters, was correlation. This was primarily due to our interest in motor control and muscular correlations in movement. Having said that, clustering can also be done using other criteria such as Euclidian distances. We attempted clustering using this criterion and did not obtain similar results (figure not included). This does not of course invalidate our results as different clustering patterns can be obtained when different criteria are applied. We would also argue that correlation is a stronger indicator of signal similarity. Two high amplitude signals that show closely varying trajectories are likely to show high correlation, but be separated by a big Euclidian distance as even small shifts would give rise to high distance values.
